# Cytotoxic Effect of Nano Fast Cement and ProRoot Mineral Trioxide Aggregate on L-929 Fibroblast Cells: an *in vitro* Study

**DOI:** 10.30476/DENTJODS.2021.87208.1239

**Published:** 2022-03

**Authors:** Mohammad Reza Nabavizadeh, Fariborz Moazzami, Ahmad Gholami, Vahid Mehrabi, Yasamin Ghahramani

**Affiliations:** 1 Dept. of Endodontic, School of Dentistry, Shiraz University of Medical Sciences, Shiraz, Iran; 2 Biotechnology Research Center, School of Pharmacy, Shiraz University of Medical Sciences, Shiraz, Iran

**Keywords:** Mineral Trioxide Aggregate, Cytotoxicity, Endodontics, Cement

## Abstract

**Statement of the Problem:**

Endodontic materials that are placed in direct contact with living tissues should be biocompatible. The cytotoxicity of Nano Fast Cement (NFC) compared to ProRoot Mineral Trioxide Aggregate (ProRoot MTA) must be evaluated.

**Purpose:**

This *In vitro* study aimed to assess the cytotoxic effects of NFC in comparison to ProRoot MTA on L-929 mouse fibroblast cells.

**Materials and Method:**

In this animal study, L-929 mouse fibroblast cells were grown in Dulbecco's Modified Eagle's medium (DMEM) supplemented with 10% fetal bovine serum (FBS) in an atmosphere of 5% co_2_/95% air at 37 C̊. A total of 10^4^ cells from the fourth collection were plated in each well of a 96-well micro-titer plate. Materials were mixed according to the manufacturer’s instruction and placed into the related plastic molds with 5 mm diameter and 3 mm height. After 24 hours and a complete setting, the extracts of the tested materials were produced at six different concentrations and placed in the related wells. Cells in DMEM served as the negative control group. DMEM alone was used as the positive control group. Methyl-thiazoltetrazolium (MTT) colorimetric assay was conducted after 24, 48, and 72 hours. The absorbance values were measured by ELISA plate reader at 540 nm wavelength. Three-way analysis of variance, post-hoc Tukey, LSD, and independent t-test were used for the statistical analyses using SPSS software, version 16.0.

**Results:**

There was no statically significant difference between MTA and NFC in cell viability values at different concentrations and different time intervals (*p*= 0.649). Viability values were significantly decreased after 72 hours, but there was no significant difference between the first and second MTT assays (*p*= 0.987). Cytotoxicity significantly increased at concentrations higher than 6.25 µɡ/ml.

**Conclusion:**

Cytotoxicity depends on time, concentration, and cement composition. There was no statistically significant difference between NFC and MTA concerning their cytotoxic effects on L-929 mouse fibroblast cells.

## Introduction

Mineral Trioxide Aggregate (MTA) was developed at Loma Linda University in 1993. It was firstly used as a root-end filling material, but the material has also been employed in apexogenesis, apexification, root canal perforation repair, and pulp capping procedures [ 1 ]. MTA is considered the "gold standard" among many endodontic types of cement owing to its ability to promote the regeneration of periodontal ligament (PDL) and hard tissue formation [ 2 ]. Several *in vitro* and *in vivo* studies have reported that MTA is biocompatible [ 3 - 5 ] and has a lower toxicity than superEBA and amalgam when it is used as retro filling material [ 6 ]. The minimum level of cytotoxicity makes MTA the best choice in many treatment procedures [ 7 - 9 ]. It also provides a good seal [ 10 - 12 ] and has antimicrobial activities [ 13 - 14 ]. However, MTA has some disadvantages such as long setting time [ 13 ], tooth discoloration [ 15 ], difficult handling [ 16 ], and high cost. An endodontic cement should ideally have a relatively short setting time to avoid being washed out by saliva and body fluids [ 17 ]. In some cases, more than one treatment session is needed to place the final restoration for the tooth in which MTA has been applied. Several new bioceramic materials were introduced that seem to have shorter setting times than MTA. Biodentine is a nano-particle calcium silicate-base cement with excellent mechanical properties and biocompatibility. It has a significantly shorter setting time (10-12 minutes) and better handling features compared to ProRoot MTA [ 18 ]. Biodentine is a great alternative to MTA in some endodontic procedures.

To overcome the inadequacies of the ProRoot MTA, a new nano-particle calcium silicate-based cement named Nano Fast Cement (NFC) (Sanat Avaran Vista, Iran) was introduced by researchers at Shiraz University. NFC has practically the same chemical composition as ProRoot MTA, although the size of its particles was decreased with Wet Stirred Media Milling (WSMM) for 15 hours, and setting time and handling features were enhanced to similar levels as Biodentine [ 19 ]. 

Endodontic materials that are placed in direct contact with living tissues should be biocompatible [ 20 ]. The purpose of the present experiment was to evaluate the cytotoxicity of NFC compared with ProRoot MTA.

## Materials and Method

### Test materials and sample preparation

Test materials used in this study were ProRoot MTA white-colored formula (Dentsply Tulsa Dental, Tulsa, Oklahoma, USA) and NFC (Sanat Avaran Vista, Iran). After sterilization with an ultraviolet beam, the materials were mixed according to the manufacturer's guidelines under aseptic conditions. Freshly mixed materials were placed into the plastic molds with 5-mm diameter and 3-mm height, and then incubated at 37˚C and 95% humidity for 24 hours. After complete setting, extracts of the materials were produced as follows: 5 ml of complete Dulbecco's Modified Eagle Medium (DMEM; Sigma Chemical Co., St. Louis, MO, USA) containing 10% Fetal Bovine Serum (FBS; Gibco, Grand Island, NY, USA) was mixed with 1 mg of each test material in sterile vials. The mixture was incubated at 37˚C at 100% relative humidity for 72 hours. The medium was then drawn off and filtered using sterile filters at 0.22 µm (Millipore; Billerica, MA, USA). The extracts were serially diluted 1:2 with complete DMEM to achieve a total of six concentrations of each extract (neat, 1/2, 1/4, 1/8, 1/16, 1/32).

The cells in DMEM served as the negative control group. DMEM alone, in 96 well tissue culture plates, were used as the positive control group.

### Cell cultures

L-929 mouse fibroblasts were grown in 75 cm^2^ flasks in DMEM containing 10% FBS, 10000 units of penicillin-G/ml, 10 mg of streptomycin/ml, and 200 mM of L-glutamine (Sigma chemical co. St. Louis, MO, USA) in an atmosphere of 5% co_2_/95% air at 37˚C. Cells were collected by washing with serum-free DMEM before being treated with 5 ml trypsin (0.1%) 0.1ml EDTA (0.01%) solution in phosphate-buffered saline for 7-10 minutes. Cells from the forth collection were plated in a 96-well plate at a density of 10^4^ cell per well and allowed to attach for 24 h to a complete DMEM.

### Metabolic assay

Methyl-thiazoltetrazolium (MTT) colorimetric assay was used for the determination of the cytotoxicity of the tested cements [ 21 ]. After 24 hours, complete DMEM was removed and 100 µl of the extracts of the experimental materials were placed in each well. After 24, 48, and 72 hours of incubation, 25µl of MTT (Sigma-Aldrich Co., St. Louis, MO, USA) stock solution was added to each well, and the plate was incubated for 4 hours. Then, dimethylsulphoxide was added to each well to solubilize the formazan crystals, and the absorbance was determined at 540 nm using an ELISA plate reader (PowerWaveTM X52, BioTek Instruments Inc., Potton, UK). Four wells were assigned to each experimental group and six wells to the control groups. At 24, 48, and 72 hours, an MTT assay was conducted on the relative plate. The cell viability was defined as the percentage of mean OD value of each cement compared with the optical density values of the negative control groups. The cell viability value of more than 90% was defined as non-toxic, 60-90% and 30-60% indicated mild and moderate toxicity, respectively, and the cell viability value of less than 30% was defined as toxic [ 22 - 24 ].

### Statistical analysis

All the analyses were performed using SPSS software, version 16.0. Three-way analysis of variance was used to assess the effect of cement type, concentration, and time on the toxicity. Post-hoc Tukey, LSD, and independent *t* tests were used to determine the differences in cell viability values. The level of statistical significance was set at 5%.

## Results

According to the results of the three-way analysis of variance, concentration (*p*< 0.001), time (*p*= 0.03), and type of cement played an important role in cell toxicity compared to the control groups (*p*< 0.001). Changes in these factors caused variations in cell viability.

Tukey test revealed no statistically significant difference between MTA and NFC in cell viability (*p*= 0.649). MTA was significantly distinct from the positive (*p*= 0.003) and negative (*p*< 0.001) control groups. There was a statically significant difference between NFC and the control groups with respect to cell viability values (*p*< 0.001). 

 LSD test was used to assess the effect of time on cell viability. The results showed that there was a statically significant difference between the third MTT assay (72 hours) with the first (24 hours) (*p*= 0.021) and second MTT assays (48 hours) (*p*= 0.022) in cell viability values. No statically significant difference was found between the first and second MTT assay values (*p*= 0.987). Cell viability decreased in the third MTT assay in both of the tested cements.

Cell viability values at 1/16 and 1/32 concentrations did not differ significantly (*p*= 0.911). Furthermore, cell viability values at neat, 1/2, 1/4, and 1/8 concentrations were almost similar (*p*= 0.802). There was a significant difference in cell viability between these two groups. Cell viability increased at lower cement concentrations.

**Figure 1:**
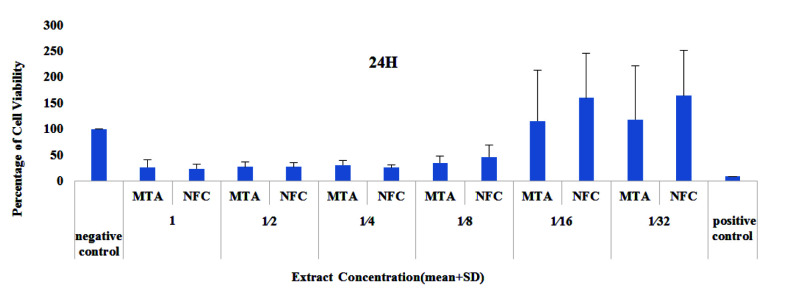
Percentage of cell viability following exposure to different concentrations of the cements (mean±SD)

 As shown in Table 1 and Figure 1, MTT assay showed that 24 hours after exposure, MTA and NFC were highly toxic at neat and 1/2 concentrations, moderately cytotoxic at 1/8 concentration and non-cytotoxic at concentrations of 1/16 and 1/32. At 1/4 concentration, MTA showed moderate toxicity, while NFC revealed severe toxicity. Independent *t* test showed that cell viability in the NFC group at 1/32 (*p*= 0.044) and 1/16 (*p*= 0.045) concentrations were significantly higher than the negative control group.

**Table 1:** Percentage of cell viability following exposure to different concentrations of the cements after 24, 48 and 72 hours

Concentration	24 hours		48 hours		72 hours	
	MTA	NFC	MTA	NFC	MTA	NFC
1	26.4	23.1	21.8	23.9	12.6	9.9
1/2	26.8	26.4	20.3	23.9	13.4	12.9
1/4	30.2	25.2	27.8	26.3	13.2	11.4
1/8	34.8	45.6	34.6	40.3	21.8	22.1
1/16	115.3	159.9	75.2	140.5	62.3	72.3
1/32	118.3	163.9	103.08	161.5	74.3	81.8

**Figure 2:**
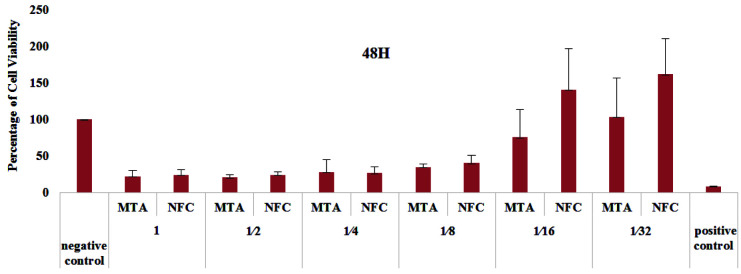
percentage of cell viability following exposure to different concentrations of the cements (mean±SD)

After 48 hours, both MTA and NFC at concentrations of 1/4, 1/2, and 1 were highly cytotoxic. They were moderately cytotoxic at 1/8 concentration. MTA revealed mild toxicity at 1/16 concentration, while NFC was not cytotoxic at this concentration. At 1/32 concentration, both cements were not cytotoxic. Furthermore, NFC provoked cell proliferation at 1/32 (*p*= 0.009) and 1/16 (*p*= 0.021) concentrations. Also, significant cell proliferation was noted at 1/32 concentration in the MTA group (*p*= 0.046) (Figure 2) compared with the negative control group (Table 1 and Figure 2).

**Figure 3:**
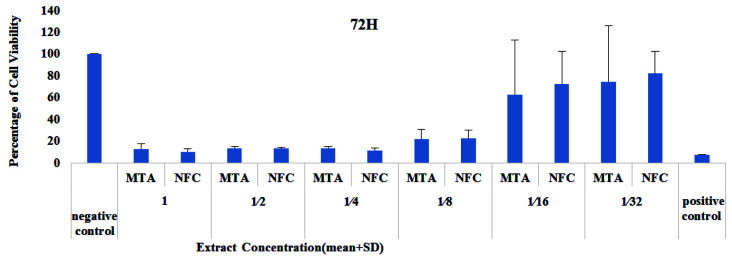
Percentage of cell viability following exposure to different concentrations of the cements (mean±SD)

After 72 hours, MTA and NFC were absolutely cytotoxic at concentrations higher than 1/16. Both of the cements revealed low toxicity at 1/16 and 1/32 concentrations. After 72 hours, cell viability was lower than the negative control group for both of the tested cements (Table 1 and Figure 3).

In the current study, the Tukey test was used to evaluate the effect of different concentrations on cell viability. 

## Discussion

Different methods have been employed to evaluate the cytotoxicity of dental materials. The MTT colorimetric assay is a common technique for assessing the toxicity of endodontic filling materials and endodontic cements [ 25 ]. Mitochondrion absorbs the tetrazole, and then succinate dehydrogenase enzyme converts the tetrazole to formazan. Afterward, an acidified solution is added to dissolve the formazan and create a colored solution. The absorbance, optical density (OD), of this colored solution can be measured by an ELISA plate reader at a certain wavelength [ 23 ]. This method is simple, rapid, and highly accurate [ 7 ]. Moreover, MTA is a hydrophilic material that releases various ions after contact with fluids, and these ions can affect the intracellular enzymes [ 6 ]. For this reason, MTT method, which evaluates the activity of the mitochondrial dehydrogenase enzyme, was chosen. Different studies have used different cell lines such as mouse gingival fibroblasts, human pulp stem cells, human endothelial cells, and so on to assess the cytotoxicity of endodontic materials [ 7 , 22 , 24 ]. Established laboratory mouse L-929 gingival fibroblasts were used, which are recommended by the International Standard Organization for primary cytotoxic evaluations [ 6 ]. To reproduce the in vivo situation, primary cell strains derived from vital human tissues are necessary.

As we mentioned earlier, MTA is the gold standard among many bioceramic cements, and its biocompatibility has been proven by many studies [ 3 - 6 ]. NFC is a bioceramic cement with nanoparticles. It contains calcium oxide, silicon dioxide, zirconium dioxide (opacifier), aluminum oxide, magnesium oxide, sulfur trioxide, phosphorus pentoxide, titanium dioxide, and carbonic acid. The size of these particles was decreased via milling due to Sanaee *et al*. method [ 19 ] tried to construct a cement with a shorter setting time (15 minutes) and simple handling compared with MTA.

In this study, a significant difference between the third MTT assay (72 hours) with both first (24 hours) and second (48 hours) MTT assays was noted. Cell viability decreased significantly after 72 hours (72 hours< 48 hours=24 hours). Saberi *et al*. [ 26 ] evaluated the cytotoxicity of several endodontic cements on stem cells of the human apical papilla. They reported that cell viability in the MTA group decreased over time. It was reported that the maximum cell viability at 24 hours belonged to the MTA group [ 24 ]. The viability of L-929 fibroblasts decreased after 48 hours. The results of our study are in line with the results of Saberi *et al*.[ 26 ] and Ghuddosi *et al*.[ 24 ], who found that longer exposure of the target cells to the toxic elements such as bismuth released from MTA and the decrease in the amount of nutrients in the culture medium after 72 hours were possible reasons for reduction of cell viability over time [ 27 - 28 ]. 

 De Deus *et al*. [ 7 ] reported an increase in the viability of endothelial cells exposed to MTA after 72 hours. Another study [ 22 ] assessed the cytotoxicity of MTA on the human pulp stem cells. They concluded that the viability of the stem cells exposed to MTA increased over time. Different target cells and different concentrations of the cements used in those two studies can explain the controversy in the results. In the latter study [ 22 ], cell density inside each well was much less (3000 cells per well) than our study. As a result, cell growth conditions were more favorable, which could lead to subsequent cell proliferation over time.

We found that cytotoxicity was dose-dependent. Cytotoxicity of the cements increased at higher concentrations. This is consistent with another study [ 24 ], which assessed the cytotoxicity of neat, 1/2, 1/10, 1/100 concentrations of MTA as well as Jaberiansari *et al*.’s study [ 22 ]. Higher concentrations of calcium silicate base cements could create a high pH environment. This alkaline pH could destroy the cell membranes and intracellular enzymes [ 29 ]. Furthermore, target cells were exposed to much more amount of cytotoxic ingredients such as bismuth. This could explain the difference in cell viability values at different concentrations. Ribeiro *et al*. [ 8 ] evaluated the cytotoxicity of MTA and Portland cement at different concentrations. Cell viability remained unchanged after exposure to different concentrations of MTA. They used a different method (single-cell gel) and different target cells (Chinese hamster ovary) for cytotoxicity assessment.

Our study compared the cytotoxic effect of MTA and NFC at six different concentrations. There was no statistically significant difference in cell viability between the tested cements at various concentrations. Viability values were highest in the NFC group at 1/32 concentration. Statistical analysis of the OD values revealed an increase in cell viability and proliferation in the NFC group at 1/32 and 1/16 concentrations after 24 and 48 hours. Also, a significant increase in cell viability in comparison to the negative control group was observed in the MTA group at 1/32 concentration after 48 hours. Jaberiansari *et al*. [ 22 ] reported significant cell proliferation in the MTA and CEM cement (BioniqueDent, Tehran, Iran) group after 48 hours, which is consistent with our results. Those findings indicated that some chemical elements released from the tested cements, such as calcium ions, can provoke cell proliferation. One study showed the important role of calcium ions on the survival of mesenchymal stem cells [ 30 ]. Calcium has a signaling ability and can up-regulate the cell function [ 31 - 32 ]. Moreover, calcium silicate base cements can act as a scaffold for cell attachment and subsequent cell proliferation.

This study had some potential limitations. MTT assay, which was used for assessing the cell viability of the fibroblast cells, cannot evaluate apoptosis and cell necrosis. In addition, the human tissue environment and defense mechanism can affect the cell response to the cements. Therefore, more animal and in vivo studies are needed to investigate the exact cytotoxicity of the NFC.

## Conclusion

The MTA and NFC had a similar effect on L-929 mouse fibroblasts. Both cements were able to induce cell proliferation at certain concentrations and specific times. 

## Conflict of Interest

The authors declare that they have no conflict of interest.

## References

[1] Abdullah D, Ford TP, Papaioannou S, Nicholson J, McDonald F ( 2002). An evaluation of accelerated Portland cement as a restorative material. Biomaterials.

[2] Perez AL, Spears R, Gutmann JL, Opperman LA ( 2003). Osteoblasts and MG‐63 osteosarcoma cells behave differently when in contact with ProRoot™ MTA and White MTA. Int Endod J.

[3] Koh ET, McDonald F, Ford TR, Torabinejad M ( 1998). Cellular response to mineral trioxide aggregate. JOE.

[4] Mitchell PJ, Ford TP, Torabinejad M, McDonald F ( 1999). Osteoblast biocompatibility of mineral trioxide aggregate. Biomaterials.

[5] Zhu Q, Haglund R, Safavi KE, Spangberg LS ( 2000). Adhesion of human osteoblasts on root-end filling materials. JOE.

[6] Keiser K, Johnson CC, Tipton DA ( 2000). Cytotoxicity of mineral trioxide aggregate using human periodontal ligament fibroblasts. JOE.

[7] De Deus G, Ximenes R, Gurgel‐Filho ED, Plotkowski MC, Coutinho‐Filho T ( 2005). Cytotoxicity of MTA and Portland cement on human ECV 304 endothelial cells. Int Endod J.

[8] Ribeiro DA, Sugui MM, Matsumoto MA, Duarte MA, Marques ME, Salvadori DM ( 2006). Genotoxicity and cytotoxicity of mineral trioxide aggregate and regular and white Portland cements on Chinese hamster ovary (CHO) cells in vitro. Oral Surg Oral Med Oral Pathol Oral Radio Endod.

[9] AlAnezi AZ, Jiang J, Safavi KE, Spangberg LS, Zhu Q ( 2010). Cytotoxicity evaluation of endosequence root repair material. Oral Surg Oral Med Oral Pathol Oral Radiol Endod.

[10] Lee SJ, Monsef M, Torabinejad M ( 1993). Sealing ability of a mineral trioxide aggregate for repair of lateral root perforations. JOE.

[11] Aqrabawi J ( 2000). Endodontics: Sealing ability of amalgam, super EBA cement, and MTA when used as retrograde filling materials. Brit Dent J.

[12] Torabinejad M, Watson TF, Ford TP ( 1993). Sealing ability of a mineral trioxide aggregate when used as a root end filling material. JOE.

[13] Torabinejad M, Hong CU, Ford TP, Kettering JD ( 1995). Antibacterial effects of some root end filling materials. JOE.

[14] Estrela C, Bammann LL, Estrela CR, Silva RS, Pécora JD ( 2000). Antimicrobial and chemical study of MTA, Portland cement, calcium hydroxide paste, Sealapex and Dycal. Braz Dent J.

[15] ( 2013). Tooth discoloration induced by endodontic materials: a literature review. Dent Traumatol.

[16] Johnson BR ( 1999). Considerations in the selection of a root-end filling material. Oral Surg Oral Med Oral Patho Oral Radio Endod.

[17] Piedad N, Zuleta F, Velasquez P, Vicente-Salar N, Reig JA ( 2014). άH-Dicalcium silicate bone cement doped with tricalcium phosphate: characterization, bioactivity and biocompatibility. J Material Sci Mater Med.

[18] Laurent P, Camps J, About I ( 2012). Biodentine induces TGF‐β1 release from human pulp cells and early dental pulp mineralization. Int Endod J.

[19] Sanaee M , Danesh Manesh H, Janghorban K, Sanaee R, Kooshesh L, Ghahramani Y, et al ( 2019). The influence of particle size and multi-walled carbon nanotube on physical properties of mineral trioxide aggregate. IOP Science.

[20] Zhou HM, Shen Y, Wang ZJ, Li L, Zheng YF, Häkkinen L, et al ( 2013). In vitro cytotoxicity evaluation of a novel root repair material. JOE.

[21] Abbaszadegan A, Gholami A, Ghahramani Y, Ghareghan R, Ghareghan M, Kazemi A, et al ( 2016). Antimicrobial and cytotoxic activity of Cuminum cyminum as an intracanal medicament compared to chlorhexidine gel. Iran Endod J.

[22] Jaberiansari Z, Naderi S, Tabatabaei FS ( 2014). Cytotoxic effects of various mineral trioxide aggregate formulations, calcium-enriched mixture and a new cement on human pulp stem cells. Iran Endod J.

[23] Mosmann T ( 1983). Rapid colorimetric assay for cellular growth and survival: application to proliferation and cytotoxicity assays. J Immunomethod.

[24] Ghoddusi J, Afshari JT, Donyavi Z, Brook A, Disfani R, Esmaeelzadeh M ( 2008). Cytotoxic effect of a new endodontic cement and mineral trioxide aggregate on L929 line culture. Iran Endod J.

[25] Gorduysus M, Avcu N, Gorduysus O, Pekel A, Baran Y, Avcu F, et al ( 2007). Cytotoxic effects of four different endodontic materials in human periodontal ligament fibroblasts. JOE.

[26] Saberi EA, Karkehabadi H, Mollashahi NF ( 2016). Cytotoxicity of various endodontic materials on stem cells of human apical papilla. Iran Endod J.

[27] Camilleri J, Sorrentino F, Damidot D ( 2015). Characterization of un-hydrated and hydrated bioaggregate and mta angelus. Clin Oral Investig.

[28] Yoshino P, Nishiyama CK, Modena KC, Santos CF, Sipert CR ( 2013). In vitro cytotoxicity of white MTA, MTA Fillapex® and Portland cement on human periodontal ligament fibroblasts. Braz Dent J.

[29] Nelson DL, Cox MM (2008). Lehninger Principle of Biochemistry.

[30] Lee BN, Lee KN, Koh JT, Min KS, Chang HS, Hwang IN, et al ( 2014). Effects of 3 endodontic bioactive cements on osteogenic differentiation in mesenchymal stem cells. JOE.

[31] Aguirre A, González A, Planell JA, Engel E ( 2010). Extracellular calcium modulates in vitro bone marrow-derived Flk-1+ CD34+ progenitor cell chemotaxis and differentiation through a calcium-sensing receptor. Biochem Biophys Res Commun.

[32] Wondergem R, Ecay TW, Mahieu F, Owsianik G, Nilius B ( 2008). HGF/SF and menthol increase human glioblastoma cell calcium and migration. Biochem Biophys Res Commun.

